# Duplication of the appendix masquerading as appendiceal tumor: a case report

**DOI:** 10.1186/s40792-023-01769-7

**Published:** 2023-10-30

**Authors:** Jumpei Shibata, Akihiro Tomida, Masaoki Hattori, Akihiro Hirata, Hiromitsu Imataki, Yukiya Orihara, Hideharu Shintomi, Keiya Aono, Motoi Yoshihara

**Affiliations:** Department of Surgery, Nishichita General Hospital, 3-1-1 Nakanoike, Tokai, Aichi 477-8522 Japan

**Keywords:** Duplication of the appendix, Laparoscopic ileocecum resection, Case report

## Abstract

**Background:**

This case report highlights the exceptional rarity of appendix duplication in adults, a condition that closely mimics appendiceal tumors, posing diagnostic challenges. The novelty of this case lies in its presentation of a Type A duplication, emphasizing the diagnostic intricacies involved in distinguishing it from other pathologies.

**Case presentation:**

We present the case of a 69-year-old male with a history of hypertension, hyperuricemia, and duodenal gastric ulcer, who presented with a positive occult blood test. Lower gastrointestinal endoscopy revealed an appendiceal orifice with atypical hyperemia and edema. Subsequent imaging and biopsy results suggested an appendiceal tumor, prompting laparoscopic ileocecal resection. Intraoperative findings revealed an unremarkable appendix, but histopathological analysis unveiled appendiceal duplication, characterized by bifurcation into two lumens within a thick serosal wall. The patient was discharged without complications.

**Conclusions:**

This case underscores the importance of recognizing appendix duplication as a rare differential diagnosis for appendiceal tumors. Surgeons should remain vigilant, especially in cases of Type A duplication, where preoperative diagnosis remains challenging. Early identification can avert potential complications and missed congenital anomalies.

## Background

Duplication within the digestive tract is exceedingly rare in adults, as more than 80% of cases manifest as acute abdominal distress or bowel obstruction before the age of two [1]. Among these occurrences, the duplication of the appendix stands as an exceptionally uncommon anomaly, with an incidence rate ranging from 0.004% to 0.009% in appendectomy specimens [2, 3]. This anomaly can often mimic other clinical conditions, such as adenocarcinoma of the colon [4], small bowel obstruction, volvulus, or intussusception [5].

Diagnosing appendiceal tumors can be a challenging endeavor due to their clinical and anatomical characteristics. In pursuit of a definitive pathological diagnosis and to mitigate the potential for malignant progression, laparoscopic ileocecal resection emerges as a viable procedure.

Within this report, we present a case detailing the duplication of the appendix, which presented a striking resemblance to an appendiceal tumor. The primary objective of this report is to heighten awareness regarding this congenital anomaly and to underscore the repercussions of overlooking a second appendix.

## Case presentation

**Fig. 1:**
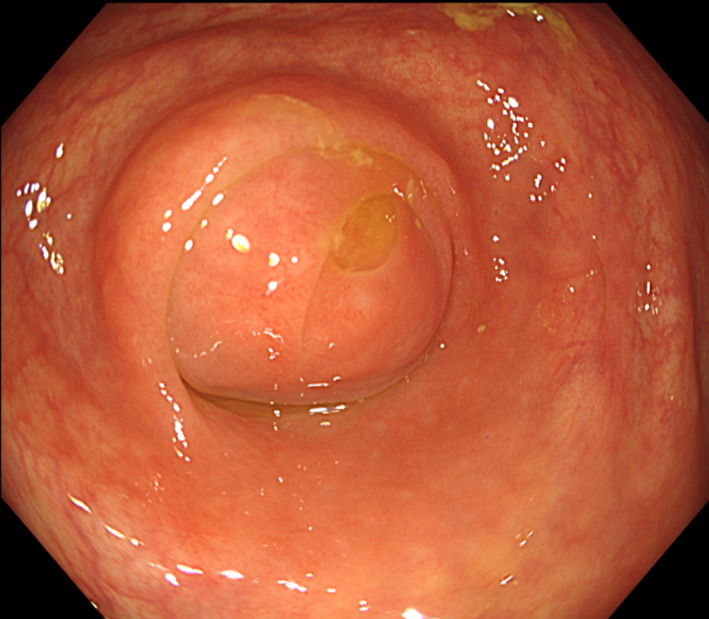
Endoscopic findings. Lower gastrointestinal endoscopy unveiled a protruding, hyperemic, and edematous appendiceal orifice

**Fig. 2:**
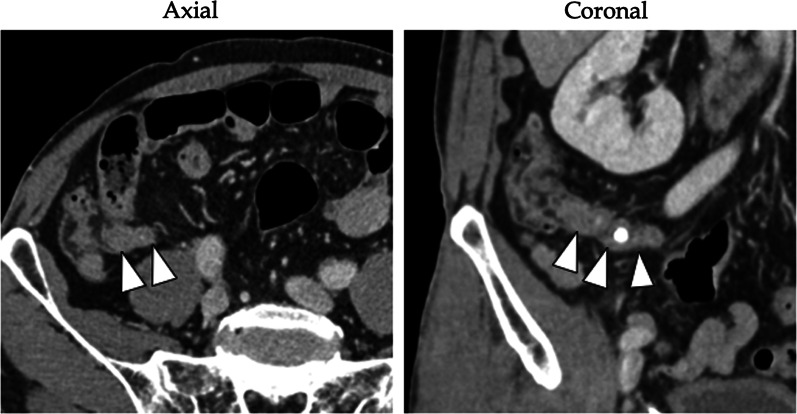
Contrast-enhanced CT scan. The CT scan revealed focal wall thickening of the appendix (indicated by arrowheads) accompanied by coprolite, but without notable enlargement of perienteric lymph nodes or apparent congenital anomalies

**Fig. 3:**
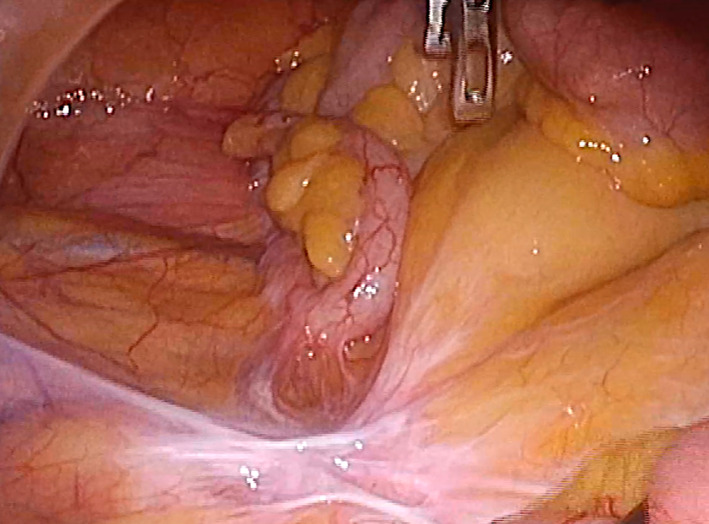
Operative findings. Laparoscopic ileocecal resection indicated no apparent abnormalities in the appendix (indicated by arrowheads)

**Fig. 4:**
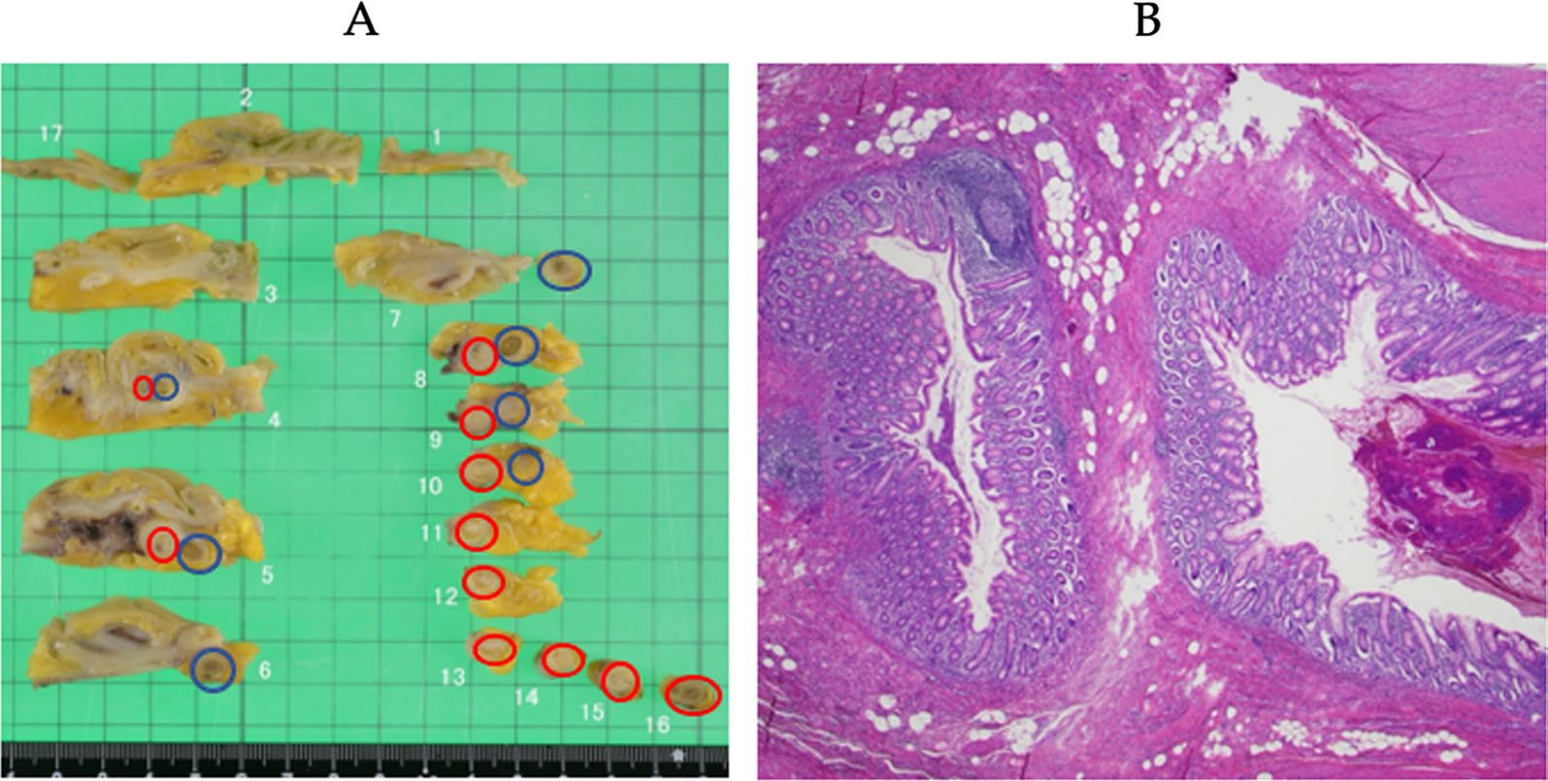
Pathological findings of the appendix. **A** Macroscopic examination of the resected specimen revealed an enlarged appendix with two lumens (represented by two circles). **B** Microscopic findings confirmed appendix duplication, with bifurcation into two lumens within a thick serosal wall. Both lumens exhibited independent true mucosa and a muscular layer

A 69-year-old male sought medical attention following a positive occult blood test result. His medical history encompassed hypertension, hyperuricemia, and duodenum gastric ulcer, which had required partial gastric resection. The patient was also a former smoker with a history of daily alcohol consumption. Physical examination yielded no noteworthy findings. Initial laboratory results indicated a serum hemoglobin level of 13.5 g/dL, a serum carcinoembryonic antigen level of 3.3 ng/dL (reference range: 0–5.0 ng/mL), and a carbohydrate antigen 19–9 level of 11.9 ng/dL (reference range: 0–37 U/mL). Lower gastrointestinal endoscopy unveiled a protruding, hyperemic, and edematous appendiceal orifice (Fig. 1). An endoscopic biopsy confirmed colon hyperplasia but fell short of delivering a definitive diagnosis. Subsequent contrast-enhanced computed tomography (CT) disclosed focal wall thickening of the appendix, alongside the presence of coprolite, without any notable enlargement of perienteric lymph nodes or apparent congenital anomalies (Fig. 2). Given a clinical suspicion of appendiceal carcinoid, mucinous neoplasm, or adenocarcinoma, the patient underwent laparoscopic ileocecal resection with regional lymph node dissection. Intraoperative findings indicated no discernible changes in the appendix (Fig. 3). Macroscopic examination of the resected specimen, however, revealed an enlarged appendix devoid of apparent abnormalities; instead, two lumens were evident throughout the appendix (Fig. 4A). Subsequent histopathological analysis confirmed the presence of an appendix duplication. Although the appendiceal base remained singular, it bifurcated into two lumens enclosed within a thick serosal wall (Fig. 4B). No evidence of malignancy was observed, and the patient was discharged seven days postoperatively without complications.

## Discussion

In this case report, we document an instance of appendix duplication that strikingly mimicked an appendiceal tumor. To the best of our knowledge, this represents a truly rare case of suspected appendiceal tumor associated with appendix duplication. The case holds instructive value for two significant reasons. First, appendix duplication can effectively mimic appendiceal tumors. Second, attaining a preoperative diagnosis is not always attainable through radiographic imaging or gross intraoperative observations.

**Fig. 5:**
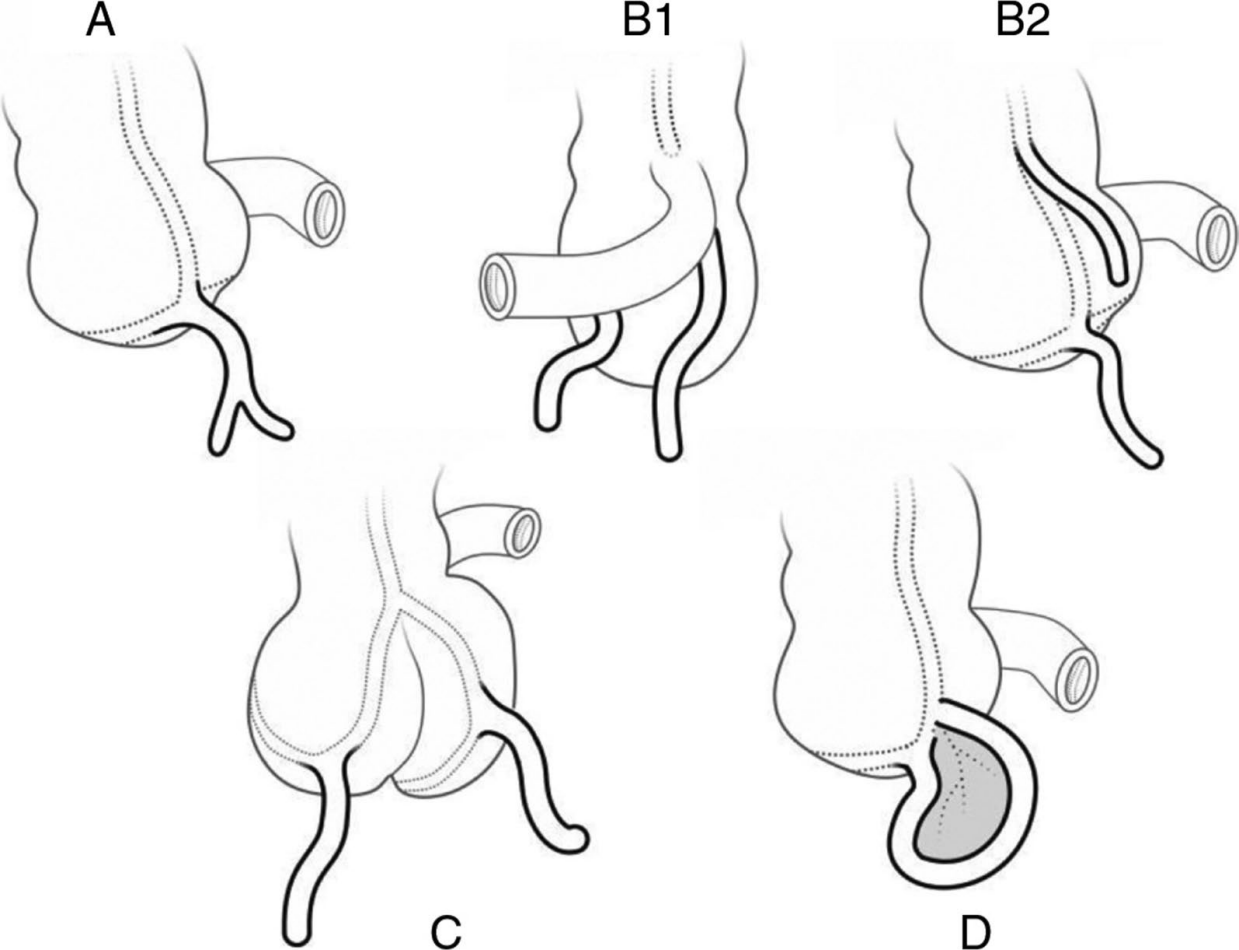
Modified Cave-Wallbridge classification. Type **A**: partial duplication of the appendix; Type **B1** (bird type): two appendices symmetrically placed on both sides of the ileocecal valve; Type **B2** (taenia coli type): one appendix in the usual position and the other along the taenia coli; Type **C**: duplication of cecum and appendix; Type **D** (horseshoe type): a single appendix with two openings in the cecum [13]

Duplication within the gastrointestinal tract is a relatively uncommon phenomenon, with appendix duplication itself being a rarity. Although its precise incidence remains challenging to ascertain, a comprehensive analysis of 50,000 appendiceal specimens yielded only two instances of duplication [2]. Despite its infrequency, the classification of appendix duplications, originally formulated by Cave [6] and subsequently modified by Wallbridge in 1963 [7], was further refined by Bierman in 2015 [8]. This taxonomy divides appendix duplications into four types, each with specific subdivisions (Fig. 5).

A. Characterized by a single cecum housing a normally localized appendix exhibiting partial duplication, which may manifest in varying degrees of incomplete duplication.
B. Comprises two complete appendices originating from a single cecum. Subdivisions within this category include:
  1. Symmetrical placement on either side of the ileocecal valve.
  2. Localization along the taenia coli.
  3. Originating in the hepatic fixture.
  4. Originating in the splenic flexure.
C. Encompasses two appendices, each emerging from its respective cecum.
D. Refers to the horseshoe appendix, which constitutes a single appendix featuring two openings in the cecum.

Type A and Type B2 duplications, which exhibit higher incidence rates (18% and 37% amongst reported cases), are typically diagnosed incidentally or when an otherwise normal or anomalous appendix presents as acute abdominal pain, with the median age of symptom are 19 or 25 years, respectively [9]. In such instances, the removal of both appendices as part of routine practice is advised to prevent “recurrent appendicitis after appendectomy”. Conversely, anomalies of Type B1 and C appendices are often detected in infants or children due to their frequent association with other intestinal, genitourinary, or skeletal malformations [10]. Although Type D duplication is extremely rare, its characteristics are similar with Type A and Type B2, without congenital concurrent abnormalities. Our case aligns with Type A duplication and is remarkable for its incidental detection, which raised suspicions of a tumor rather than acute appendicitis.

In light of the suspicion of an appendiceal tumor, our patient underwent planned laparoscopic ileocecal resection. Conducting a diagnostic appendectomy followed by further resection based on pathology findings is a feasible approach. However, patients suspected of having appendiceal tumors often remain asymptomatic or exhibit nonspecific symptoms. Since appendiceal lesions typically present as submucosal tumors during endoscopy, colonoscopy rarely detects appendiceal abnormalities and seldom provides a diagnostic biopsy [11]. Recognition of appendiceal tumors may rely on specific colonoscopy findings, such as a smooth indentation of the cecal lumen or the appearance of a glossy, rounded, protruding mass emerging from the appendiceal orifice [12]. Based on these colonoscopy findings and the CT scan, we concluded that the risk of neoplastic involvement was relatively high, prompting us to select ileocecectomy as the definitive procedure. Furthermore, it is plausible that the specific type of duplication in our patient contributed to the erroneous preoperative diagnosis. Type A duplication is characterized by a thicker base of the appendix compared to other types, as both lumens are encapsulated within a single serosal wall.

It is paramount for any surgeon performing appendectomy to possess a comprehensive understanding of the potential etiologies. Familiarity with anatomic variations and anomalies proves invaluable in ensuring the delivery of appropriate surgical treatment and facilitating discussions with colleagues and patients. In most reported cases, discovery occurs during surgery with a presumed diagnosis of acute appendicitis. The preoperative detection of appendiceal duplication remains challenging, particularly in cases mirroring Type A duplication, such as our own.

## Conclusions

This report highlights a case of appendix duplication that strikingly resembled an appendiceal tumor. Although exceedingly rare, the recognition of appendix duplication holds critical significance for surgeons. Failure to identify such anomalies may yield unexpected outcomes, including the oversight of a second appendix and associated congenital anomalies. Preoperative radiographic and ultrasonographic imaging, along with gross intraoperative findings, do not consistently yield a definitive diagnosis. Therefore, surgeons should remain vigilant regarding this anomaly to mitigate potential confusion with intra-abdominal structures.

## Data Availability

All data supporting our findings are contained within manuscript.

## Abbreviation

CT: Computed tomography
